# CMV Pancreatitis in an Immunocompromised Patient

**DOI:** 10.1155/2021/8811396

**Published:** 2021-02-19

**Authors:** Jaffer Ahmad, Najia Sayedy, Raghavendra Sanivarapu, Jagadish Akella, Javed Iqbal

**Affiliations:** ^1^Nassau University Medical Center-Department of Medicine, USA; ^2^Nassau University Medical Center-Department of Pulmonary and Critical Care Medicine, USA

## Abstract

**Introduction:**

Cytomegalovirus (CMV) is a common double-stranded DNA (dsDNA) virus affecting a large majority of the world's population. In immunocompetent patients, CMV infection can range anywhere from an asymptomatic course to mononucleosis. However, in the immunocompromised patient, prognosis can be deadly as CMV can disseminate to the retina, liver, lungs, heart, and GI tract. We present a case of CMV pancreatitis afflicting an immunocompromised patient. *Case Summary*. A 45-year-old Hispanic female with no past medical history presented to the emergency department (ED) for three days of abdominal pain associated with nausea, vomiting, and diarrhea. ED vitals showed a sepsis picture with fever, tachycardia, low white blood cell (WBC) count with bandemia, and CT scan showing acute pancreatitis, cholelithiasis, gastritis, and colitis. The patient denied alcohol use and MRCP showed no stone impaction. Sepsis protocolled was initiated for biliary pancreatitis, and the patient was admitted to the medicine floors with appropriate consulting services. Over the course of admission, the patient responded poorly to treatment and had a steady decline in respiratory status. She tested positive for HIV with a severely depressed CD4 count (42 cells/McL) and high viral load (1,492,761 copies/ml) and started on appropriate prophylactic antibiotics and HAART therapy. The patient was moved to the Medical Intensive Care Unit (MICU) after acute respiratory failure secondary to ARDS requiring mechanical ventilation with initiation of ARDS protocol. The patient was hemodynamically unstable and required vasopressor support. Hospital course was complicated by melena which prompted an esophagogastroduodenostomy (EGD) with biopsy yielding CMV gastritis. Serum CMV viral load was also found to be positive along with an elevated lipase level, indicative of pancreatitis. Despite initiation of ganciclovir, the patient continued to have refractory hypoxia despite full ventilatory support and proning. Unfortunately, the patient was deemed too unstable for transfer to an ECMO facility. She eventually succumbed to respiratory failure. *Discussion*. CMV is a Herpesviridae virus that is prevalent among more than half of the world's population. Its effects range from no presenting symptoms to respiratory failure depending on immune status. CMV more commonly affects the retina, lungs, liver, and GI tract; however, in rare cases, it is known to affect the pancreas as well. Other more common causes of pancreatitis were ruled out during the progression of this patient, and an elevated lipase with high CMV viral load points towards CMV pancreatitis.

**Conclusion:**

This is one of only a few reported cases of CMV pancreatitis and warrants further study due to the massive prevalence of CMV in the entire world's population. Our case demonstrates the extent of dissemination of CMV in a severely immunocompromised patient by showing clear cut pancreatitis secondary to said viral infection with exclusion of other possible causes. Our hope is that clinicians will change their practice to include a more scrutinized study into causes of pancreatitis especially in their immunocompromised patients.

## 1. Introduction

Cytomegalovirus (CMV) is a common double-stranded DNA (dsDNA) virus found in approximately 59% of the population [1]. In the immunocompetent patient, it has a self-limiting course; however, in the immunocompromised patient, CMV infection can often have a grave prognosis [2]. CMV in immunocompromised patients is aggressive and can disseminate to multiple organs causing disease including but not limited to retinitis, hepatitis, esophagitis, colitis, and pneumonitis. The retina is the most common site for disseminated CMV to proliferate and cause inflammatory responses; however, CMV has the potential to spread to any organ [3, 4]. We present a rare case of CMV pancreatitis in a 45-year-old immunocompromised woman from El Salvador.

## 2. Case Presentation

**Figure 1:**
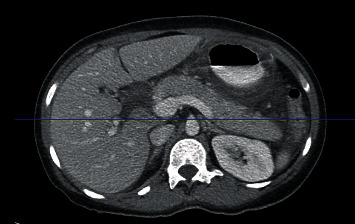
CT abdomen image obtained in Emergency Department indicating findings consistent with pancreatitis.

A 45-year-old Hispanic female with no significant past medical history presented to the emergency department with three-day history of abdominal pain, nausea, vomiting, and diarrhea. Abdominal pain was described as a severe, burning sensation in the epigastrium radiating to her back, exacerbated by po intake and associated with fevers, nonbloody, nonbilious emesis, and enumerable episodes of nonbloody diarrhea. Vitals on admission were as follows: Temp 101.1F BP 104/67 mmHg P 101 RR 17 SpO2 98% on ambient air. On physical exam, the patient had tenderness to palpation over her right upper quadrant. Laboratory results revealed a WBC 5.32, low lymphocyte count (11%), bandemia (10%), ALT of 26 U/L, AST of 39 U/L, Alkaline Phosphatase of 90 U/L, and a lipase count 3 times the level of normal (153 U/L). A CT abdomen/Pelvis revealed acute pancreatitis, cholecystolithiasis, gastritis, colitis, and moderate ascites (Figure 1).

On the medicine floors, the patient was initially started on ciprofloxacin, metronidazole, and cefepime for acute biliary pancreatitis and colitis, and coverage was broadened to meropenem and amikacin for persistent fevers (101-102F), worsening leukocytosis and worsening clinical symptomatology of epigastric pain, diarrhea, nonbloody, nonbilious emesis, and dyspnea with exertion. Despite approximately 6 L fluid resuscitation with normal saline, the patient remained hypotensive. An MRCP revealed a single 0.8 cm stone in the fundus of the gallbladder yet no bile duct impaction, making biliary pancreatitis less likely. A week into her admission, a screening HIV test was found to be positive with a CD4 of 42 cells/McL.

**Figure 2:**
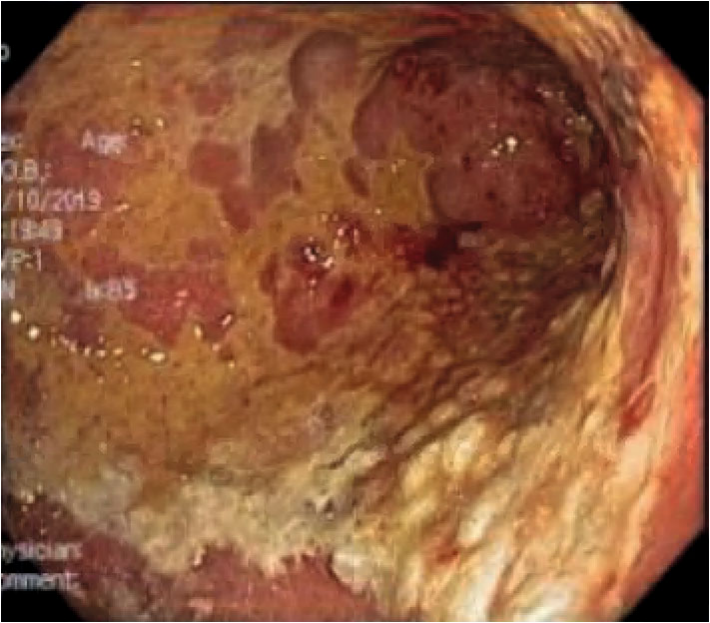
EGD image of body of stomach showing ulceration secondary to CMV.

**Figure 3:**
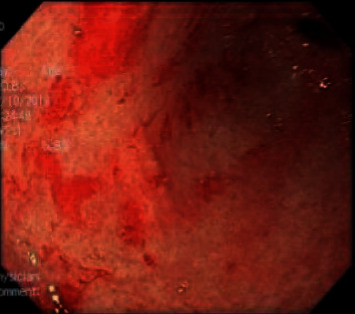
EGD image of antrum of stomach showing ulceration secondary to CMV.

With her hemodynamics steadily deteriorating likely secondary to respiratory distress secondary to Pneumocystis jiroveci, the pneumonia patient was started on TMP/SMX and systemic steroids, and MAC prophylaxis and Descovy were also initiated. However, despite these measures, the patient had a declining respiratory status. A chest X-ray showed diffuse pulmonary haziness, and a CT thorax revealed diffuse ground-glass opacities consistent with ARDS. The patient was hypoxemic on a nonrebreather mask, and her saturation continued to fall on high flow oxygen. A bronchoscopy was performed due to high suspicion of pneumocystis pneumonia (PCP). Bronchoalveolar lavage (BAL) cytology was negative for malignant cells as was PCP; however, aspergillus was cultured. During the hospital course, the patient began to desaturate to 70% on 70% FiO2 via Hi-Flow nasal cannula. Her PaO2/FiO2 ratio was <100 indicating severe ARDS, requiring intubation, and was subsequently transferred to MICU service. Chest X-ray obtained postintubation compared with earlier X-rays correlated with ARDS. MICU admission was complicated by melena prompting endoscopic workup. Esophagogastroduodenoscopy (EGD) revealed nodular ulcers in the esophagus, extensive gastric ulcers with exudates, and hemorrhage throughout the stomach with EGD biopsies revealing severe active chronic inflammation with epithelial cells with eosinophilic inclusions consistent with cytomegalovirus infection (Figures 2 and 3). Furthermore, serum CMV viral load was more than 2 million and Ganciclovir was initiated. Ophthalmology was consulted for possible CMV retinitis which was ruled out. Abdominal ultrasound revealed moderate ascites and cholecystitis.

Despite ARDS protocol initiation including paralytic initiation and prone positioning, the patient had refractory hypoxia. The patient was deemed too unstable for Extracorporeal Membrane Oxygenation (ECMO). Despite initiation of Continuous Renal Replacement Therapies (CRRT), her renal function and severe metabolic acidosis failed to improve. Despite the exhausted efforts of the MICU team, the patient became severely hypoxemic, bradycardic, and eventually passed away. Autopsy was declined by the family.

## 3. Discussion

CMV, a member of the Herpesviridae family, is a double-stranded DNA virus and replicates itself inside the host cell nucleus. It uses its host to package more infectious products and dense bodies which enumerate in quantity and eventually lyse the cell and spread throughout the host. CMV-infected cells have a textbook histological footprint, often presenting with “Owl-eyes” inclusions within the nucleus of the cell [3].

In immunocompetent patients, CMV is usually asymptomatic or can present with flu-like symptoms and possibly enlarged lymph nodes and/or spleen. For such patients, it is usually self-limiting and does not progress any further. Immunocompromised patients, however, are afflicted with a more severe and disseminating infection. The two biggest populations of immunosuppressed patients infected with CMV are transplant recipients and HIV/AIDs infected patients. It is known that CMV in HIV patients typically affects part or all of the GI tract, the most prominent being colitis, esophagitis, and gastritis. Our patient presented similarly with multiple imaging modalities confirming each of these. The EGD performed revealed multiple irregular ulcers in the esophagus and a vast blanket of exudative ulcers in the stomach that made the tissue friable and prone to bleeding [4, 5].

Pancreatitis is an atypical finding in CMV infection. However, reports reveal that it is known to afflict these groups of immunocompromised patients, including those on chronic steroid or immunosuppressive therapy and patients with systemic lupus erythematosus [5]. Our patient presented with diagnostic findings consistent with acute pancreatitis including abdominal pain, lipase three times the upper limit of normal within 3 days of symptom onset, and imaging (both CT abd/pelvis and abdominal ultrasound) findings consistent with acute pancreatitis. Further lab work during the patient's admission showed an elevated Anti-1-Antitrypsin level and an elevated amylase, also consistent with acute pancreatitis.

There are numerous causes of pancreatitis, the most common of which are alcohol (variable consumption period of 4-7 drinks per day) and gallstones. The patient denied any alcohol use and though the MRCP showed a single 0.8 cm gallstone in the fundus of the gallbladder; it also showed no stone impaction within the common bile duct, making the diagnosis of biliary pancreatitis less likely. Furthermore, certain medications have been known to cause pancreatitis including but not limited to amiodarone, enalapril, losartan, isoniazid, and sulfonylureas. Our patient endorsed no known medical history or regular medication [6].

Certain factors are identified that could have aided towards a stronger case of pancreatitis. Although the evidence strongly suggests CMV pancreatitis, a positive biopsy or autopsy would have provided a definitive diagnosis. Biopsy of the pancreas could not be performed due to the patient's critical status and the family of the patient declined autopsy, so confirmation could not be confirmed. The immune status of the patient was not discovered until later into the development and decline of the patient. Late into her admission, she revealed her history of rape by multiple different men (5 years ago) while attempting to cross into US border; she never revealed her rapists' identity or sought medical attention afterwards as she feared her undocumented status would be revealed. CMV was tested for much later on, and although valganciclovir therapy only slows down the progression of the disease, it was started only after CMV infection was confirmed. The monitoring of pancreatic enzyme levels regularly could have mapped out the status of the patient's pancreatitis and given a better picture of just what phase she was in based on the levels. The patient's family refusal for autopsy hindered the possibility of pancreatic biopsy to confirm infection of the pancreas with CMV.

## 4. Conclusion

Due to the prominence and its effect on more than half of the world's population, we encourage clinicians to further the study of CMV and the reach of its dissemination. Our case accurately portrayed the effect of CMV in an immunocompromised patient and revealed how devastating a common Herpesviridae virus can be to a host with a suppressed immune system. By encouraging this study, we hope to help clinicians recognize the commonalities within the pattern of symptoms and signs for both CMV infection and pancreatitis. This is one of only a few cases of CMV pancreatitis found in a literature review, and it is our goal that we can use this study to aid in the early diagnosis and treatment of disseminated CMV.
